# Evaluation of surface passivating solvents for single and mixed halide perovskites†

**DOI:** 10.1039/d2ra04278a

**Published:** 2022-10-11

**Authors:** Mehmet Derya Özeren, Áron Pekker, Katalin Kamarás, Bea Botka

**Affiliations:** Institute for Solid State Physics and Optics, Wigner Research Centre for Physics Konkoly Thege u. 29-33 H-1121 Budapest Hungary kamaras.katalin@wigner.hu bea.botka@wigner.hu; Department of Physical Chemistry and Materials Science, Faculty of Chemical Technology and Biotechnology, Budapest University of Technology and Economics Műegyetem rkp. 3 H-1111 Budapest Hungary

## Abstract

Surface passivation is one of the commonly used approaches to reduce the density of defects on the surfaces and interfaces hindering the performance and stability of perovskite optoelectronic devices. Although surface passivation leads to performance improvement for the targeted devices, details of the complex intermolecular interactions occurring between the molecules and perovskites are not entirely known. Here, we investigated a variety of commonly used solvents in the post-processing of perovskites by using photoluminescence (PL) spectroscopy on single and mixed halide perovskites (MAPbI_3_, MAPbBr_3_ and MAPb(Br_0.5_I_0.5_)_3_). Our results show that solvents with medium and low Gutmann donor and acceptor numbers provide PL intensity increase for both single halide perovskites by passivating the surface defect sites. Among the single halide perovskites, MAPbBr_3_ is more attracted to hydrogen bonding solvents, in contrast to MAPbI_3_ that is preferred by Lewis bases. This halide selective attraction also has an influence on the mixed-halide composition. Identifying these interaction mechanisms provides new insights into passivating the surface of perovskites for future device design.

## Abstract 2

Various surface passivating solvents with different functional groups were used to investigate solvent–perovskite interactions. The identification of the underlying mechanisms provides insight for new surface passivation strategies.

## Graphical abstract

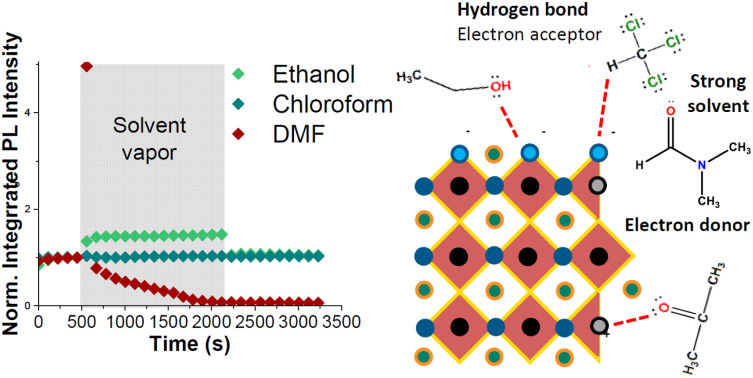

## Introduction

Organic–inorganic halide perovskites have been playing a significant role in photovoltaic applications in the past years owing to their compositional flexibility, solution processability and easier adaptability to scale up production methods. Single junction solar cells have already reached 25.7% conversion efficiency owing to defect passivation and crystallization strategies.^1^ Higher device performances have been initially obtained with small adjustments in the nucleation-growth mechanism of lead halide perovskites such as use of excess precursors and/or modification of precursor concentration,^2–5^ addition of secondary solvent and/or using alternative solvents,^6–9^ which lead to control of the film formation dynamics and morphology. Although a variety of approaches are used to modulate the nucleation-growth mechanism, improved film properties are mainly observed due to the reduction of defect density. The most common defect types encountered in the MAPbX_3_ based perovskites that influence the frontier orbitals of the Pb–X network (and, consequently, both film photoluminescence and device performance) are uncoordinated lead and uncoordinated halide ions.^10–12^ Grain boundaries and surfaces are rich with these type of defects, and also the place where the structural deterioration starts by the adsorption of water and oxygen.^13,14^ Bulk and surface passivation of these defect states are the main requirements to boost the performance and the stability of perovskite devices. For this purpose, a variety of techniques have been applied in recent years, often involving multistep processes with various solvents and solutions.^15^ These techniques have been optimized towards practical purposes and the required macroscopic parameters (polarity, solvent composition *etc.*) were chosen accordingly.

In this paper, we take another approach and concentrate on microscopic processes between the perovskite layer and pristine solvents in the vapor phase. The starting point in describing the effect of solvents on perovskites is based on Lewis acid–base interactions.^16^ Such interactions play a principal role in both perovskite deposition and passivation studies. The affinity of a Lewis base to a Lewis acid is generally described by the Gutmann donor number,^17–19^ and it allows estimation of the strength of the dative or coordinate bonding. Besides the experimentally determined donor number, Gutmann also introduced an acceptor number for Lewis acids,^20^ that include the proton and thus can be applied to hydrogen bonds as well. We will use these parameters to understand the effect of commonly used solvents on the surface of single and mixed-halide perovskites (MAPbI_3_, MAPbBr_3_ MAPb(Br_0.5_I_0.5_)_3_) in post-processing under continuous illumination by following their steady-state photoluminescence. With these experiments, we model both environmental effects and targeted passivation. We study the interaction of uncoordinated lead atoms with Lewis donors and that of uncoordinated halides with Lewis acceptors (including hydrogen-bond donors).

## Experimental

All chemicals used are from commercially available sources without any further purification.

### Perovskite thin film preparation

Undoped silica substrates are cleaned by ultrasonic bath sonication for 10 minutes in each solvent acetone, ethanol and distilled water, then plasma cleaned for 15 seconds to form hydrophilic surfaces. 1 M of MAPbX_3_ precursor solutions (PbX_2_ + MAX, where X = I, Br) are prepared in dimethylformamide (DMF) in a N_2_ filled glovebox. After stirring overnight, equivalent volumes of these solutions are mixed to form 1 M of MAPb(Br_0.5_I_0.5_)_3_ perovskite precursor solution and stirred for 30 min. To prepare perovskite thin films, 10 μL of precursor solutions are statically dropped onto prepared silica substrates, then a spin coating process is initiated at 2000 rpm for 9 seconds, followed by 4000 rpm for 30 seconds. When the spin coating process is completed, samples are transferred to a hot plate and annealed at 100 °C for 30 minutes.

### Photoluminescence measurements of thin films

**Fig. 1:**
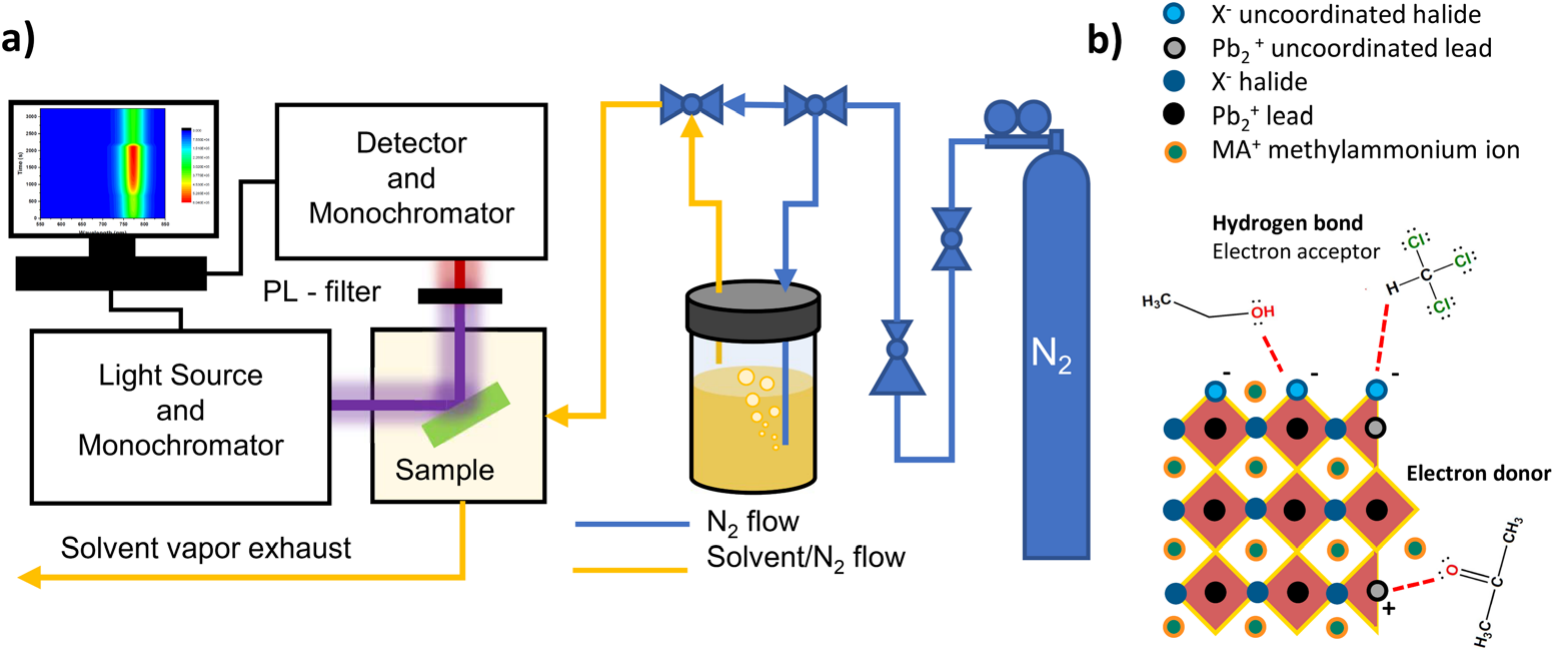
Illustration of the *in situ* PL measurement design (a) and the uncoordinated halide and lead interactions with donor and acceptor-type solvent molecules (b).

Photoluminescence of perovskite samples is recorded by a Horiba Jobin Yvon Nanolog Fluorimeter with 0.5 seconds integration time at 430 nm illumination wavelength and 3 mW cm^−2^ illumination intensity in all measurements unless otherwise mentioned. Prepared perovskite thin films are put into a sealed cryostat in a N_2_ filled glove box and transported to the spectrometer, then N_2_ flow connections are set up for the cryostat and it is kept under N_2_ flow in the dark for a couple of minutes before the beginning of the measurement. Solvents are introduced into the sample chamber by continuous N_2_ bubbling during the designated exposure period. The schematic of the PL measurement is illustrated in Fig. 1a. Measurements consist of a total of 30 cycles divided into three sections 5 scans for initial inspection, 15 scans during solvent exposure and 10 scans for recovery period with one-minute delay between cycles. Comparison of three samples with varying PL intensity variations during the initial inspection period and the prolonged illumination on the samples are shown in Fig. S1.† Exposure to solvent under dark is completed with the same exposure time as under illuminated conditions.

### UV-visible measurements

Perovskite solutions are recorded by an Ocean Optics fiber optics spectrometer under laboratory conditions of 23 °C with 30% relative humidity. Samples are prepared in a glove box, then placed into cuvettes filled with the selected solvents. The cuvettes are lightly sealed to prevent the evaporation of solvents during the measurement. The solutions are kept for 2 hours dark to observe the spectral changes over time caused by the dissolution of surface species. The schematics of the UV measurement is illustrated in Fig. S6.†

### Infrared measurements

Powdered perovskites are placed onto a molybdenum crystal to measure the attenuated total reflection infrared (ATR-IR) spectra using a Bruker Tensor 37 FTIR instrument in the 800–4000 cm^−1^ mid-infrared region with an average of 256 scans. Exposure of the sample to water is conducted in the same way as with photoluminescence measurements and with the same exposure unit.

Far-infrared spectra are measured using a Bruker IFS66v spectrometer with 6 μm mylar beamsplitter and an average of 128 scans. All measurements are carried out with 4 cm^−1^ resolution.

## Results and discussion

Molecules that can donate and accept lone electron pairs are defined as Lewis bases and acids, respectively, and the transfer of a pair of electrons from an occupied orbital of a base to an unoccupied orbital of an acid is called a Lewis acid–base reaction. Uncoordinated Pb^2+^ in perovskites acts as a Lewis acid and interacts with electron donors,^21,22^ while uncoordinated halides behave as Lewis bases. Hydrogen bond donor molecules can behave as Lewis acids and accept electrons from uncoordinated halides. We evaluated the Lewis acid–base interactions of commonly used solvents in perovskite post-processing with halide and lead defects on the surfaces and interfaces based on their electron donor–acceptor abilities by using donor and acceptor numbers given in Table S1.†*In situ* PL spectroscopy allows us to examine the effects of the ongoing passivation processes almost instantly, on a scale of seconds, by causing PL intensity increase or decrease related to defect passivation or formation during the exposure and recovery periods. We start this section with the effect of electron donor solvents interacting with uncoordinated Pb^2+^, then we examine the effects of electron acceptor solvents on uncoordinated X^−^ in single halide perovskites. These will be followed by the evaluation of the effects of both electron donor and acceptor solvents on mixed halide perovskites. Possible interactions of the solvent molecules with the uncoordinated atoms on the perovskite surfaces are illustrated in Fig. 1b. As methylamine defects have no effect on the PbI-based frontier orbitals and therefore their influence on either the PL or the device performance is less, we do not take these interactions into account.

### Interactions in single halide perovskites

**Fig. 2:**
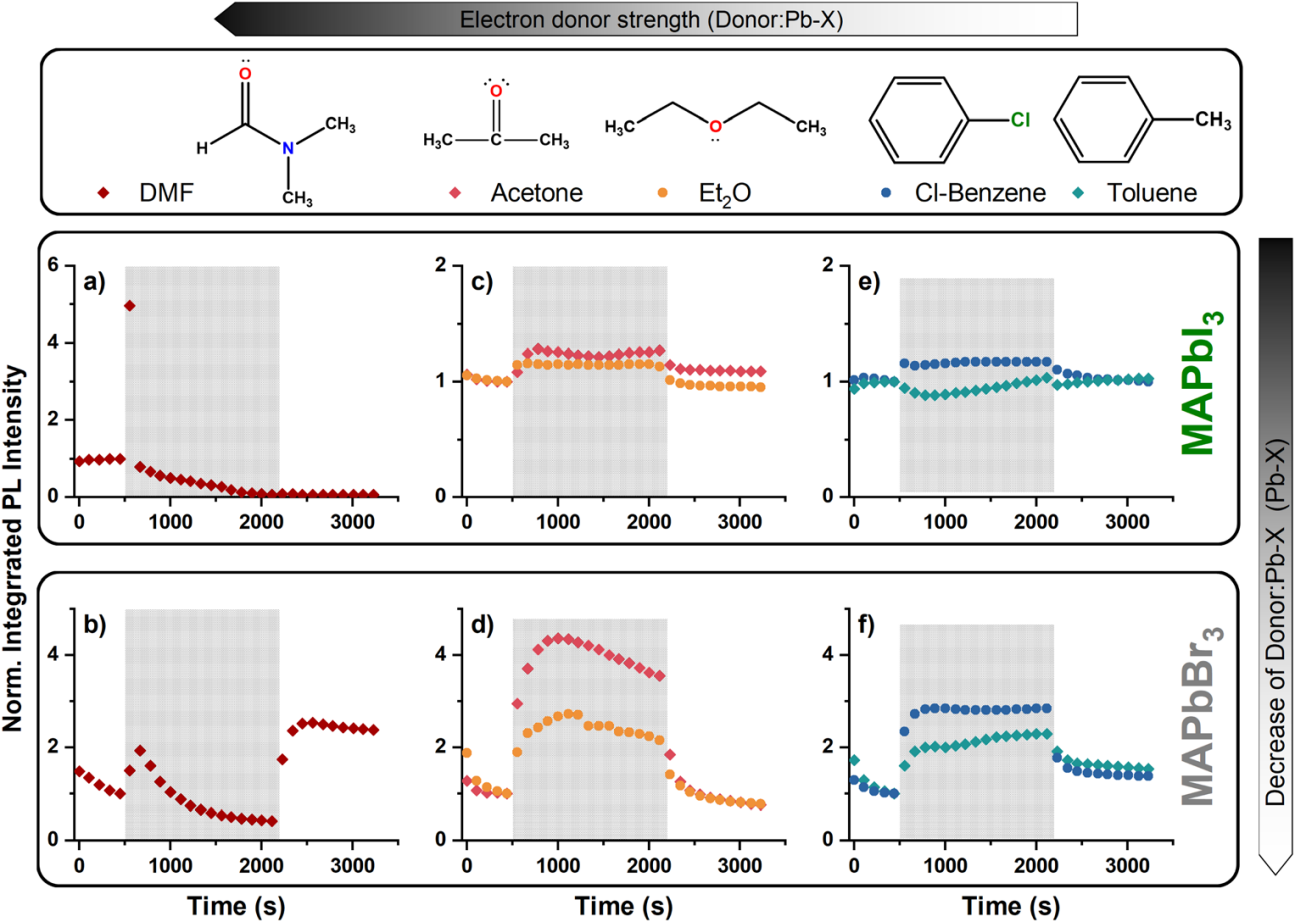
Effects of various electron donor solvents on the PL spectra of MAPbI_3_ and MAPbBr_3_. PL spectra changes of MAPbI_3_ and MAPbBr_3_ during exposure to DMF (a, b), diethyl ether/acetone (c, d) and chlorobenzene/toluene (e, f) are presented in the first and second row of the graph. The orange patterned zone (between 445–2115 seconds) represents the solvent exposure period, and the left and right side of the patterned zone denotes N_2_ environment in the continuous measurement process. The PL intensities are integrated between 700–850 nm and 470–600 nm emission wavelength for MAPbI_3_ and MAPbBr_3_, respectively, and normalized to the integrated intensity value of the last measurement (5^th^ scan) in the initial N_2_ environment (0–445 seconds). The reversible PL intensity increase observed during the interaction with medium-low electron donor ability solvents indicates temporary passivation of Pb^+^ defects in both MAPbBr_3_ and MAPbI_3_.

Fig. 2 shows the changes of the MAPbBr_3_ and MAPbI_3_ PL intensity during exposure to electron donating solvents. Full PL mapsfor are shown in Fig. S3.† Here DMF is a strong solvent in Tutantsev's classification,^23^ reacting with all components of the perovskite, therefore the interactions go beyond surface passivation. During the exposure of the surface of MAPbI_3_ to DMF, quenching of the PL is observed after a quick PL intensity increase at the beginning of the exposure period (Fig. 2a). Such increase, in the case of moderate solvents, usually indicates surface defect passivation; however, for this strong solvent it is the consequence of the formation of a supersaturated state caused by the high solubility of the Pb–I cage. The PL intensity increase is accompanied by a simultaneous blue shift of the peak position (Fig. S3 and S4†). Recently a similar PL intensity increase and shift was observed as the result of instant formation and growth of nanocrystallites during the annealing step of the spin-coating process and consequent quantum confinement.^24^ Based on these results, we assume that the first small amount of DMF forms a liquid–solid phase resulting in nanocrystals; more solvent gradually dissolves these crystals and a surface reconstruction takes place,^25^ decreasing the PL signal. In the case of MAPbBr_3_ (Fig. 2b), the PL intensity loss is taking place during a longer period, due to lower solubility of the perovskite in DMF. During the initial interaction with DMF, the PL intensity of MAPbBr_3_ does not increase as that of MAPbI_3_ does and its peak position is stable during the whole exposure period (Fig. S3†). The main difference observed in the PL spectra between MAPbBr_3_ and MAPbI_3_ is the instant recovery of the MAPbBr_3_ PL intensity when the N_2_ environment is reestablished. The intensity of MAPbBr_3_ nearly doubles compared to its initial value, indicating a surface reformation similar to “solvent vapor annealing”.^26–28^ Such a reformation process can also be considered for MAPbI_3_, however, that process requires annealing or a longer drying period. The recovery of the original black color of MAPbI_3_ dried under air is shown in Fig. S4.† This recovery behavior, and the overall difference between MAPbBr_3_ and MAPbI_3_ in this subsequent period of solvent interaction, confirms that the tendency to form coordinate bonding is higher for MAPbI_3_.

The decrease of the electron donating ability weakens the forming coordinate bond and provides an interaction without the disruption of the perovskite structure during the solvent exposure period. MAPbI_3_ and MAPbBr_3_ surfaces exposed to diethyl ether/acetone show PL intensity increase during the exposure period by the passivation of uncoordinated Pb^2+^ defects (Fig. 2c and d). When the N_2_ environment is reestablished, the effect disappears, and the PL intensity returns to its initial level on both perovskites. Interestingly, chlorobenzene, having one of the lowest donor numbers, also provides similar passivation effect on both MAPbI_3_ and MAPbBr_3_ (Fig. 2e and f). On the other hand, toluene, with a similar structure, selectively affects the PL intensity depending on the halide ion. The MAPbI_3_ PL intensity shows a decrease after the surface is exposed to toluene. UV-visible measurements indicate that methylammonium lead halides release halogens in toluene (Fig. S5†). Similar results were observed on MAPbI_3_ in earlier studies, and the reason of MAPbI_3_ instability is pointed out as iodine release from the structure due to its high solubility in toluene.^29,30^ Indeed, the 300 nm absorption peak growing with time coincides with that of the charge-transfer complex forming between iodine and toluene in solution.^29^ Since toluene only dissolves extrinsic ingredients in the crystal, the PL intensity changes cannot be attributed to donor–acceptor interactions.

It is worth noting here that acetone, diethyl ether and chlorobenzene cause higher PL intensity increase on MAPbBr_3_ (Fig. 2d and f) than MAPbI_3_ (Fig. 2c and e). The explanation could be the lower solubility of the bromide containing perovskite in these solvents, which can provide an advantage to passivate the defects without any surface disruption. Another, and more ideal, reason is the higher surface area of the MAPbBr_3_ than MAPbI_3_ to interact with. The SEM images in Fig. S7† show that MAPbBr_3_ has island-like crystals spread over the substrate, in contrast to the entangled morphology of MAPbI_3_, which makes the defect sites more approachable by solvent molecules. Overall, the behavior of the electron donor interactions can be influenced by two distinguishable effects, surface size and electronic interactions. The Pb–I bond, being less polar than the Pb–Br bond, is more sensitive to electron donating solvents.

Next, we will examine the hydrogen bond donor solvents that can contribute to the passivation of uncoordinated X^−^. We will regard hydrogen bond formation as electron transfer from the proton acceptor to the proton donor and utilize their acceptor number to characterize the strength of the donor–acceptor interaction. In the case of hydroxyl group containing solvents, the issue is complicated by the fact that they can behave towards Pb^2+^ as Lewis bases through their oxygen lone electron pairs. To separate the sole electron acceptor interaction, chloroform is selected as a weak hydrogen bond donor.

**Fig. 3:**
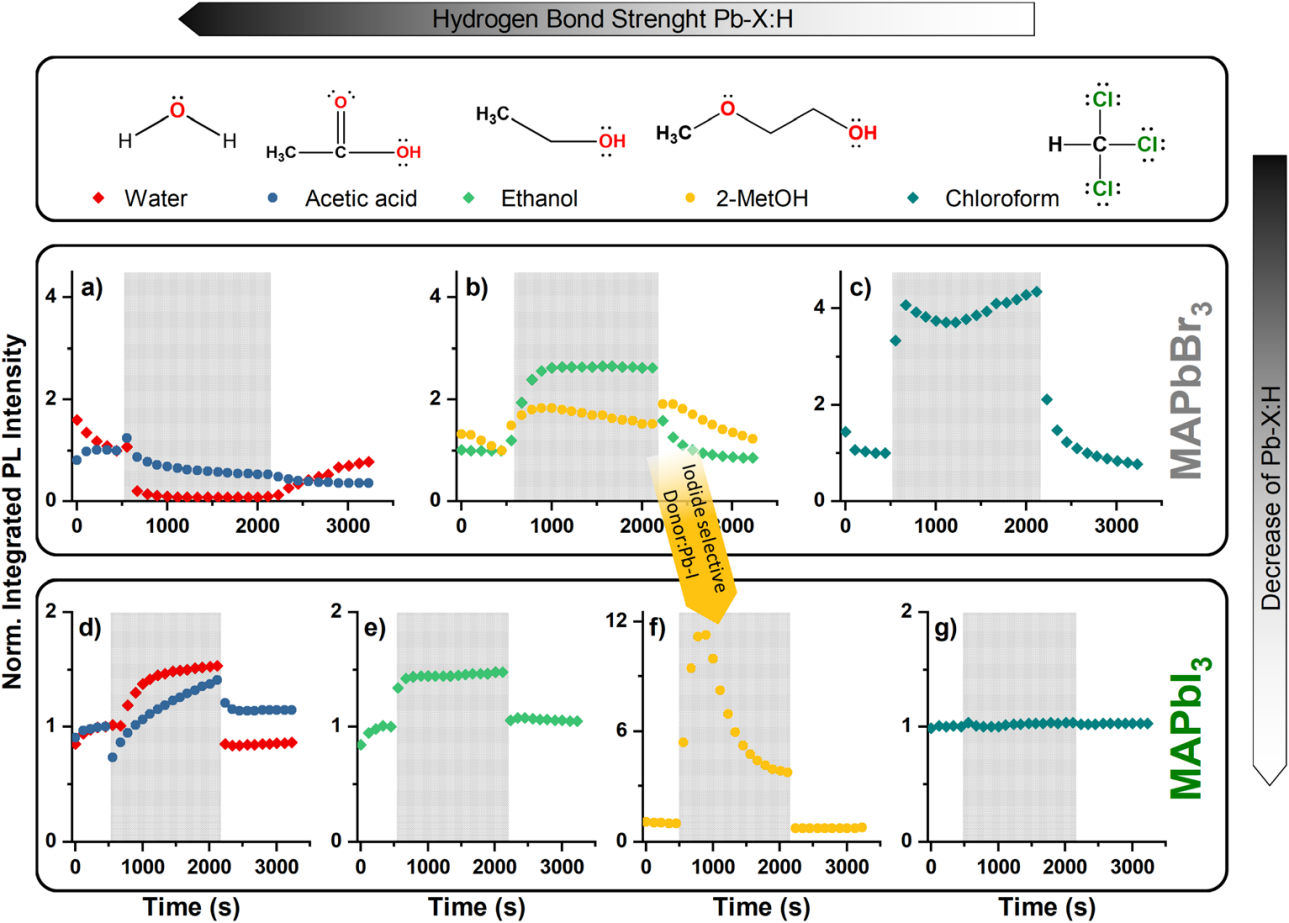
Effects of the hydrogen bonding solvents with varying acceptor ability on the PL spectra of MAPbBr_3_ and MAPbI_3_. PL spectra of MAPbBr_3_ and MAPbI_3_ are given in the first and second row of the graph and their changes by interacting with water/acetic acid (a and b), ethanol/2-MetOH (2-methoxyethanol) (c, d and e), chloroform (f and g) during the exposure period are indicated with the orange patterned zone. The left and right side of the orange patterned zone is under N_2_ atmosphere, where PL spectra after the orange patterned zone show the recoverability of the surface. The PL intensities are integrated between the values of 700–850 nm and 470–600 nm emission wavelength for MAPbI_3_ and MAPbBr_3_, respectively, and normalized to the integrated intensity value of last measurement (5^th^ scan) in the initial N_2_ environment (0–445 seconds).

Fig. 3c shows the MAPbBr_3_ PL intensity change after the surface is exposed to chloroform. The PL intensity is reaching a value four times higher than its initial intensity during the period that solvent–perovskite interactions take place, possibly by the passivation of uncoordinated Br^−^ anions on the surface. The occurring interactions are reversible and the PL intensity recovers when the atmosphere is restored. In contrast to the MAPbBr_3_ response, no such passivation effect is observed on MAPbI_3_ when the surface is exposed to chloroform (Fig. 3g and S8†). This result is in accordance with the lower electronegativity of iodine. Furthermore, the low solubility of perovskites in chloroform is proven by UV-visible spectra (Fig. S5†). As a result, PL and UV results validate the weak interaction between the chloroform and perovskites, and the tendency of the bromide anion and the proton of the chloroform to form a Lewis pair.

The interaction mechanism of hydroxyl group-containing solvents is not as straightforward as in the case of chloroform. The hydroxyl group can passivate either uncoordinated Pb^2+^ or X^−^ (due to possible Lewis acid and base properties) on the perovskite surface, which makes it elusive to interpret from which part of the hydroxyl group the PL intensity changes are originating in the spectra. As can be seen in Fig. 3b and e, the hydroxyl group of ethanol provides a decent reversible PL intensity increase on both MAPbBr_3_ and MAPbI_3_, which can result from either electron donor or acceptor interactions, or both 2-methoxyethanol (2-MetOH) has a chemical structure similar to ethanol with an additional ether functional group that behaves as an electron donor due to the lone electron pairs on the oxygen. This makes 2-MetOH a multifunctional molecule, capable to interact with both uncoordinated Pb^2+^ and X^−^ ions simultaneously. By taking advantage of its special chemical structure, we used this molecule to show that the selection of halide in perovskites dominantly affects which type of defect interacts with a passivating molecule and contributes to the PL changes. In Fig. 3b, nearly twofold PL intensity increase of MAPbBr_3_ can be seen after the surface is exposed to 2-MetOH, that is later followed by a gradual decrease until the end of the interaction period, indicating a slight disruption of the surface by the methoxy group. MAPbI_3_, on the other hand, shows significant PL intensity increase in the first couple of minutes, followed by a fast-paced decrease. This difference shows that the methoxy group affects MAPbI_3_ more significantly than MAPbBr_3_. As we already discussed earlier, the iodide ion shows higher affinity to electron donor groups and less tendency toward hydrogen-bond formation. Therefore, it is plausible to interpret that the hydroxyl group interacts selectively with perovskites based on the halide ion, where hydrogen as an electron acceptor is dominant on MAPbBr_3_ and oxygen as an electron donor is dominant on MAPbI_3_. It is also known that 2-MetOH is a good solvent for MAPbI_3_ precursor salts,^31,32^ and detecting a PL intensity response similar to the effect of DMF is reasonable (Fig. S3†). Consequently, electron donor–acceptor interactions of amphoteric hydroxyl groups with a medium donor and acceptor ability can contribute to the passivation of both lead and halide defects, and the selection of halide affects the relative strength of possible interactions. Care should be also taken when selecting or designing molecules containing multiple functional groups as surface passivating agents.

In the last part of the evaluation of hydroxyl group containing solvents, we consider water and acetic acid due to their close electron donor and acceptor numbers and the high value of the latter. As can be seen in Fig. 3a, the MAPbBr_3_ PL intensity is quenched partially or completely after a brief increase when water/acetic acid is introduced into the sample chamber, which is not surprising considering the high affinity of Br^−^ to H^+^ compared to I^−^. Higher resolution measurements are also given in Fig. S9,† where the PL intensity increase can be seen more clearly at the beginning of the interaction period. Similar to our results, PL quenching response was observed in an earlier study with water.^33^ Moreover, the intensity of MAPbBr_3_ is partially recovered only in the case of water when the sample chamber is purged by nitrogen. This indicates that acetic acid afflicts a more detrimental effect on the surface, despite the complete PL quenching caused by water. Additionally, we also tested prolonged exposure of the MAPbBr_3_ surface to acetic acid to clarify its effect on PL intensity loss, however, after a certain point the PL intensity of MAPbBr_3_ reached a steady state without further loss (Fig S10†).

The interactions of water and acetic acid with MAPbI_3_ are not as detrimental as we observed on MAPbBr_3_ (Fig. 3d and S9†). The PL intensity of MAPbI_3_ gradually increases while exposed to water. The effect is reversible, and the PL intensity nearly returns to its initial value when the atmosphere is recovered. In contrast to water, the PL intensity loss of MAPbI_3_ is distinctly observable in the first minute when the surface is exposed to acetic acid. This intensity loss later follows a gradual increase until the end of the exposure period, and it partially recovers to its original state when the atmosphere is purged. The intensity loss on MAPbI_3_ can be correlated with an instantaneous defect concentration increase on the surface by the acidic proton. The reason is that the oxygen atom (of the carbonyl and hydroxyl) should cause PL intensity increase even if it partially dissolves the surface as we experienced earlier with 2-MetOH (Fig. 3f). Overall, these results, along with the effects of water and acetic acid on MAPbBr_3_, allow us to conclude that MAPbI_3_ is more resistant to acidic interactions, and should have longer stability under humid atmosphere. Molecules having high electron acceptor ability or acidic groups should be avoided to contact with perovskite surfaces to improve the stability while designing cell structures.

Interactions of strong hydrogen bonding solvents might lead to anion exchange reactions between the perovskite and solvent molecules resulting in the formation of Pb(OH), Pb(OH)_2_, PbO.^34,35^ To evaluate possible reaction products, mid-infrared (mid-IR) measurements are taken during exposure to water of both MAPbBr_3_ and MAPbI_3_ by *in situ* ATR spectroscopy (Fig. S11†). Water is introduced by the same procedure used in the PL measurements and spectra are taken under N_2_ atmosphere except the exposure period. The original infrared peaks of both perovskites are subtracted to observe the changes during and after the water exposure. Excess water absorption is clearly observable at 1640 cm^−1^ and around 3500 cm^−1^ during the exposure period. When the environment is recovered to N_2_, the only change observed affects the N–H vibrations of both MAPbBr_3_ and MAPbI_3_ without any new bands assignable to the possible Pb-based reaction products. We have also measured the far-infrared spectra of the water and acetic acid exposed thin films to look for possible lead compounds. As can be seen in Fig. S9,† apart from the original Pb–X peaks of MAPbBr_3_ and MAPbI_3_,^36^ no new bands appear after the samples are exposed to water and acetic acid.

### Interactions in mixed halide perovskites

In this last part, we discuss the effects of the donor–acceptor interaction on the segregation of mixed halide perovskites. Mixed halide perovskites are potential candidates for several applications, *e.g.* tandem solar cells, as their band gap can be easily tuned in the range of bromide and iodide single halide perovskites (530–780 nm) by changing the stoichiometry. The ionic potential difference of bromide and iodide causes halide mismatch in the nucleation-growth stage,^37^ which makes mixed halide perovskites prone to be highly defective compared to single halide perovskites. Upon illumination, certain mixed-halide compositions become unstable and single-halide regions form. A small portion of the iodide ions migrates to the surface and grain boundaries with the assistance of defects and forms clusters at thermodynamically stable iodide-rich compositions. The excited charge carriers funnel into these newly formed, lower-bandgap regions and recombine there. This process is known as halide segregation or Hoke effect,^38^ and causes the PL spectra to be dominated by the emission of the newly formed iodide-rich phase (segregate phase), independent from the initial composition of the mixed halide perovskite. Despite the uniqueness of the segregate phase formation in mixed-halide perovskites, single-halide perovskites also suffer from similar vacancy-mediated ion migration, which causes hysteresis, and was even pointed out as one of the reasons of the degradation under prolonged illumination.^29,39^ Passivation of the defects has a significant impact on this process, because it can prevent or slow down the initial formation of the segregate phase by blocking ion migration pathways, or it can disrupt the charge funneling process by altering charge diffusion lengths (formation of electric field and dipole by the donor–acceptor interactions).^40,41^

Fundamentally, the aspects of donor–acceptor interactions that we observed on single-halide perovskites are similar in the case of mixed halide perovskites. On the PL spectra, however, we observe the interactions from the side of the minority segregate phase, not from the mixed halide phase itself. In a simplistic assumption, the donor–acceptor interactions should cause PL changes which are the mirror image of the main single halide phase interactions. Passivation of the surface defects should reduce the segregate phase and increase the mixed halide phase intensity by healing the defects. Conversely, with increased defect concentration, the halide segregation should accelerate.

**Fig. 4:**
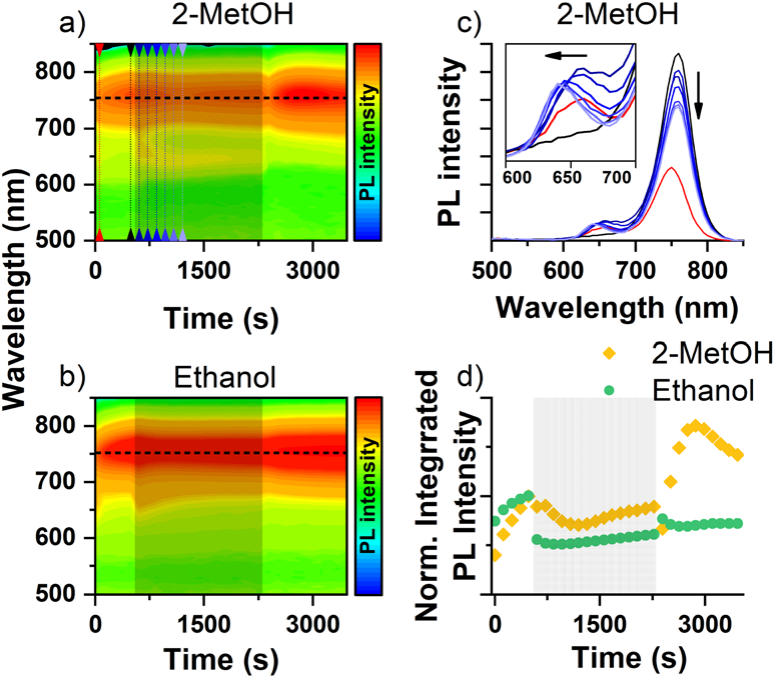
The effect of 2-MetOH and ethanol vapor on MAPb(Br_0.5_I_0.5_)_3_ mixed halide perovskites. (a and b) PL maps of MAPb(Br_0.5_I_0.5_)_3_ showing the intensity changes caused by 2-MetOH and ethanol molecules (PL intensity in logarithmic scale). The designated solvent exposure period is indicated by the light black colored zone. (c) Line graph of MAPb(Br_0.5_I_0.5_)_3_ showing the effect of 2-MetOH. The selected peaks are indicated by the colored lines on PL maps. The inset shows the mixed phase intensity changes on the selected times. (d) Integrated PL intensity changes of the segregate phase caused by the 2-MetOH and ethanol interactions. Light colored grey zone indicates the solvent exposure period.

The effect of solvent vapors can be seen in Fig. 4a and c, when the MAPb(Br_0.5_I_0.5_)_3_ surface interacts with 2-MetOH and ethanol. 2-MetOH is a good example for the effect of surface passivation because the PL intensity of the segregate phase immediately decreases (Fig. 4b and d) and the mixed phase recovers (Fig. 4b inset) when oxygen atoms donate their unshared lone pair electrons. Exposing the perovskite to other electron donor solvents, such as ethanol or acetone (Fig. S12†) results in a similar PL response. The comparison of the changes in segregate phase intensity for ethanol and 2-MetOH can be seen in Fig. 4d as well. However, the mixed phase PL intensity recovery, which is obvious in the case of 2-MetOH and other donor solvents, is barely observable here. This suggests that there may be additional mechanisms that contribute to the response observed. First, chemical alteration of the iodide-rich segregate phase can occur. As we already know from earlier results (Fig. 3f), the methoxy group is individually attracted to iodides, and we can accordingly assume that it can disperse the iodide-rich segregates on the surface. As a second possibility, dipoles can form by the donor–acceptor interactions. The newly formed surface dipole can repel generated charges to reach formed iodide segregates, which results in the reduction of the segregate phase intensity. This, in turn, forces charge carriers to recombine in the mixed phase, providing a recovery in mixed phase intensity.

The PL intensity of the segregate phase goes through a minimum and then increases again during the solvent exposure in case of 2-MetOH. This is potentially a result of two competing processes, passivation of the surface leading to the decrease of iodide pockets found there, and filling up of remaining or newly formed segregate pockets within the bulk, driven by the concentration gradient induced by the initially formed iodide segregates.

We note that in the recovery period there is an unusual maximum indicating two parallel processes, affecting the segregate phase intensity (Fig. 4d). We are not sure about the reason for this phenomenon. Similar PL response can be observed after exposure to diethyl ether and ethanol, indicating the same, albeit weaker underlying mechanism.

**Fig. 5:**
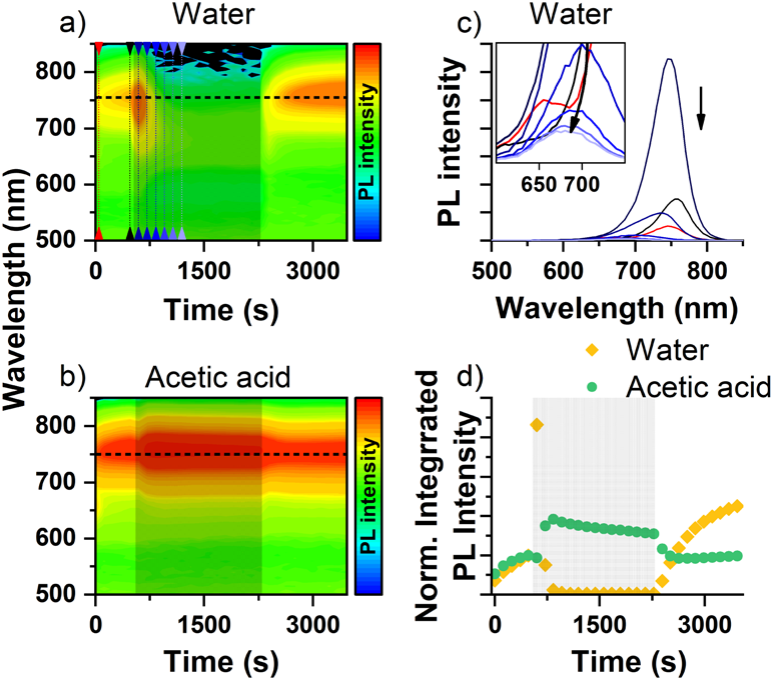
The effect of water and acetic acid vapor on MAPb(Br_0.5_I_0.5_)_3_ mixed halide perovskites. (a and b) PL maps of MAPb(Br_0.5_I_0.5_)_3_ showing the intensity changes caused by water and acetic acid molecules (PL intensity in logarithmic scale). The designated solvent exposure period is indicated by the light black colored zone. (c) Line graph of MAPb(Br_0.5_I_0.5_)_3_ showing the effect of water. The time of the selected peaks are indicated by the colored lines on PL maps. The inset shows the mixed phase intensity changes for the selected times. (d) Integrated PL intensity changes of the segregate phase caused by the water and acetic acid interactions. Light colored grey zone indicates the solvent exposure period.

Last, we are going to evaluate the effect of water and acetic acid on the mixed halide perovskites. As we mentioned earlier, water and acetic acid have relatively close donor and acceptor numbers, and we could expect a similar effect on the segregate phase intensity. The effect of both solvents on the PL spectra of MAPb(Br_0.5_I_0.5_)_3_ is given in Fig. 5, where a sharp increase of the segregate phase PL intensity can be seen immediately after water exposure is started. This is later followed by a rapid PL quenching of the segregate phase, indicating the suppression of halide segregation with a slight intensity loss of the mixed halide PL peak (Fig. 5b). In the recovery period, the segregate phase PL intensity quickly recovers, indicating that water does not cause major degradation, in line with the single halide measurements. We observed similar results in our earlier study, where the segregate phase intensity is only quenched while the perovskite surface interacts with water or humid air for K^+^ (Lewis acid) added mixed halide perovskites.^42^

Contrary to what we would expect based on its donor–acceptor properties, acetic acid causes the segregate PL intensity to increase (Fig. 5c and d). This can be explained with dipole formation on the surface. This dipole has the opposite direction than that of 2-MetOH due to their different acceptor number (Table S1†), causing the migration of generated electrons and negatively charged halides from the bulk toward the surface. In addition, we already observed immediate PL intensity loss on MAPbI_3_ when the surface is exposed to acetic acid (Fig. 3d and S9†), which coincides with our observation on the mixed halide perovskite. In case of acetic acid, we also observe two parallel processes during the solvent exposure, indicated by two maxima. This is similar to the case of 2-MetOH, just in the opposite direction.

Moreover, the PL intensity suppression by water cannot be considered the result of passivation, otherwise defect healing should result in a recovery of the mixed phase PL intensity as we observed with 2-MetOH rather than a slight loss during water exposure. It is possible that water molecules impair the funneling of the generated charges and behave as artificial obstacles on the surface and grain boundaries. The quick recovery of the segregate phase also indicates that iodide-rich regions are not significantly interrupted by water molecules in this process.

The presented results on the mixed halide sample show the complex nature of the solvent–perovskite interactions. While some part of the effects can be explained based on single halide results, the complete identification of the underlying chemical and physical processes needs more investigation.

## Conclusions

We evaluated surface interactions and their effect on the PL properties of MAPbBr_3_, MAPbI_3_ and MAPb(Br_0.5_I_0.5_)_3_ perovskites by using a variety of solvents with known donor and acceptor numbers on the Gutmann scale. Our results show that solvents with medium-low donor or acceptor numbers are optimal for surface passivation. Surface interactions highly depend on the selection of the halide in methylammonium-based lead perovskites, especially on its electronegativity. Donor functional groups such as carbonyl and ether tend to interact and bond with uncoordinated Pb^+^ ions atoms in perovskites containing iodide. Conversely, molecules with a functional group that can behave as an acceptor tend to be attracted to perovskites containing bromide. Our evaluation with the hydroxyl group molecules also indicates the same tendency towards the halide with a higher electronegativity to form a coordinate bond on the surface, which highlights the degradative contribution on MAPbBr_3_ when the acceptor number is high.

In addition, we have evaluated a mixed halide perovskite, MAPb(Br_0.5_I_0.5_)_3_, considering the halide selective tendency of functional groups to interact with the surface. Our measurements showed that donor–acceptor interactions of the solvents such as acetic acid and 2-MetOH not only contribute to the passivation of the defects on the surface, but also to the migrating species. Water (with high acceptor number), even tends to quench the segregate PL intensity rapidly when it is exposed to the mixed halide perovskite surface.

In the light of these findings, we believe that selective or halide-dependent passivation strategies can be applied effectively in future devices by designing new molecules to passivate and protect the perovskite surface.

## Author contributions

M. D. Ö., B. B. and Á. P. conceived the project. M. D. Ö. did the sample preparation, photoluminescence, infrared and UV-visible spectroscopy measurements. M. D. Ö., Á. P. and B. B. interpreted the results. All authors took part in formulating the final conclusions and writing the paper.

## Conflicts of interest

There are no conflicts to declare.

## Supplementary Material

RA-012-D2RA04278A-s001
