# Supplementary material for: Evaluation of surface passivating solvents for single and mixed halide perovskites
Source: RSC Adv. 2022 Oct 11;12(44):28853–61. doi: 10.1039/d2ra04278a (PMC9552863; doi:10.1039/d2ra04278a)
Supplement: RA-012-D2RA04278A-s001 [file RA-012-D2RA04278A-s001.pdf]

Electronic Supplementary Material (ESI) for RSC Advances.
This journal is © The Royal Society of Chemistry 2022

Supporting information for

Evaluation of surface passivating solvents for single and mixed halide perovskites

Mehmet Derya Özeren, Áron Pekker, Katalin Kamarás, Bea Botka

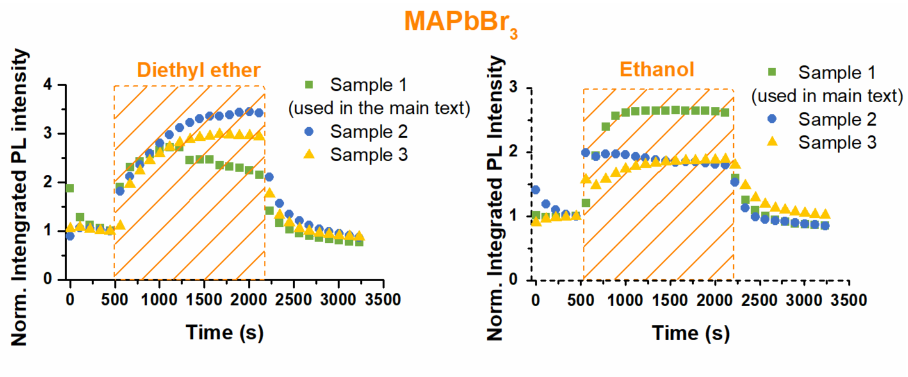

Figure S1. PL cycles for three samples of $MAPbBr_3$ exposed to diethyl ether and ethanol. Slight photoluminescence intensity variations in the initial inspection period (0-500 seconds) have no effect on the subsequent pattern during solvent exposure and the recovery period.

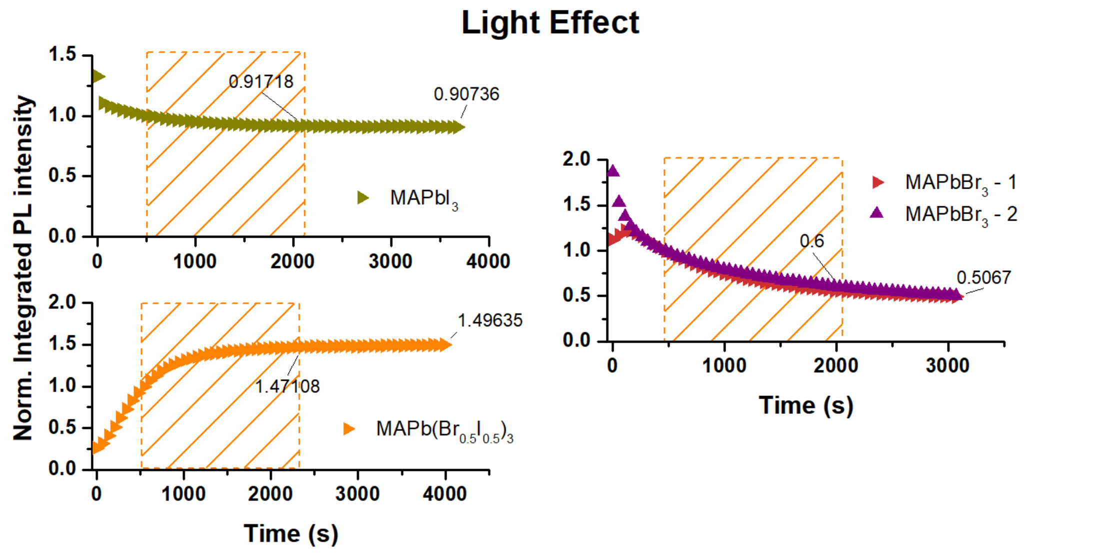

Figure S2. The effect of prolonged illumination (3 mW/cm^2 at 430nm) on the PL response of $MAPbI_3$, $MAPbBr_3$ and $MAPb(Br_{0,5}I_{0,5})_3$. While $MAPbI_3$ shows almost no degradation upon illumination and the mixed halide sample also reaches stability within the solvent exposure timeframe, the integrated PL intensity for $MAPbBr_3$ drops to 40% of its initial value. However, this process is overruled by solvent interactions as soon as the vapor is introduced and does not change qualitatively the behaviour of the measured PL intensity.

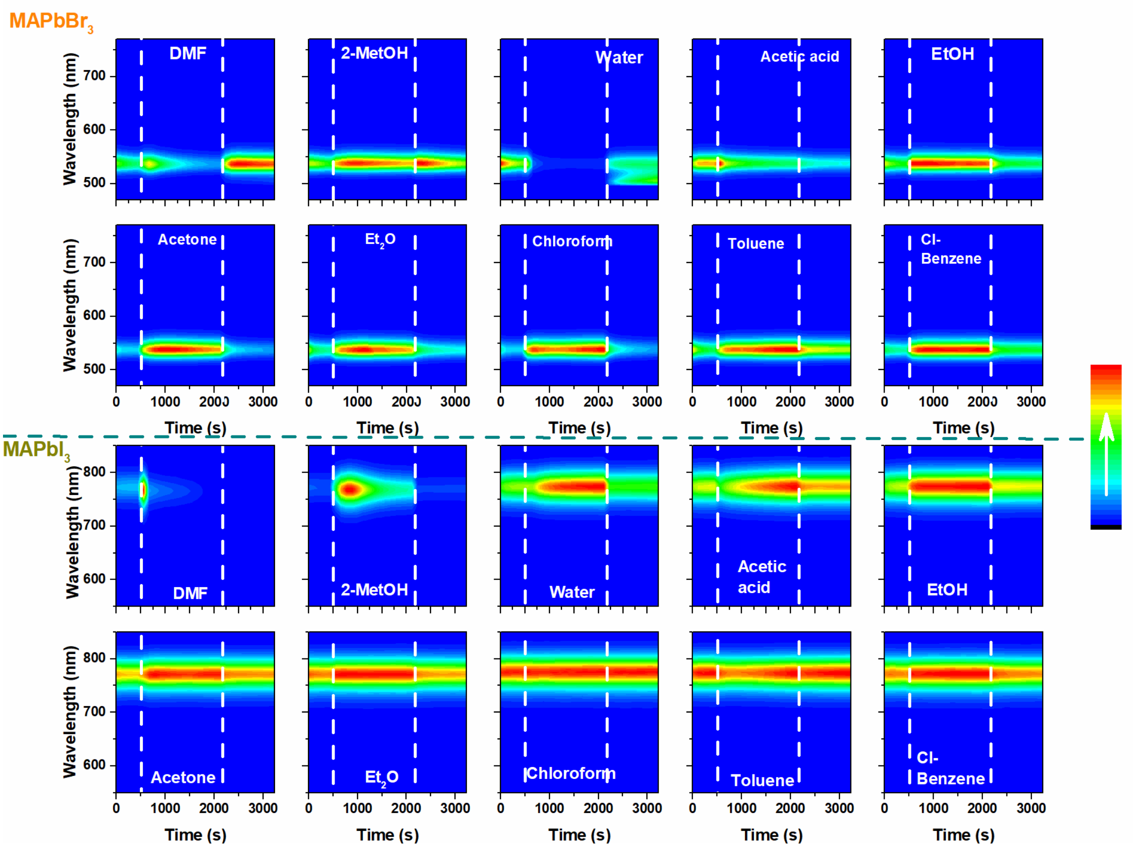

Figure S3. Illustration of all examined solvent effects on the $MAPBr_3$ (top row) and $MAPI_3$ (bottom row) PL intensity. Solvent exposure periods (445-2115 seconds) are indicated by the white dashed lines. (DMF: Dimethylformamide, MetOH: 2-methoxyethanol, EtOH: ethanol, Et_2O: Diethyl ether, Cl-benzene: Chlorobenzene)

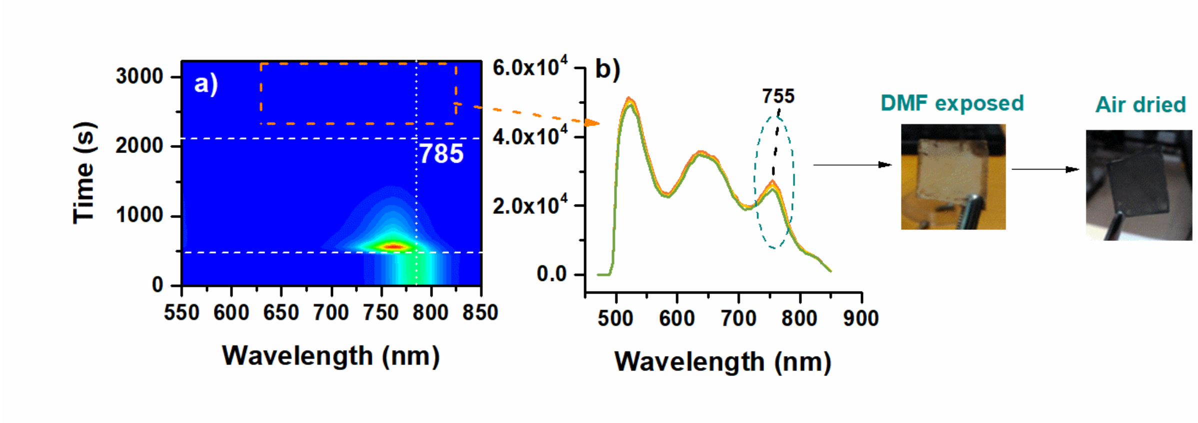

Figure S4. Lead halide solvate formation of $MAPbI_3$ on glass substrate. Effect of DMF exposure is shown on the PL map of $MAPbI_3$ (a), and its specific time frame (2228-3230 seconds) is highlighted on the line graph (b). The transparent film represents the lead halide-DMF solvate formation after the measurement period is completed, while the dark film represents the recovery of the perovskite s after the film is dried at room temperature. The initial PL position of the $MAPbI_3$ film (785nm) is shown with vertical dotted lines, and the solvent exposure period indicated by white horizontal dashed lines. The dashed elliptical circle on the line graph shows the blue shifted PL peak when the $MAPbI_3$ is exposed to DMF.

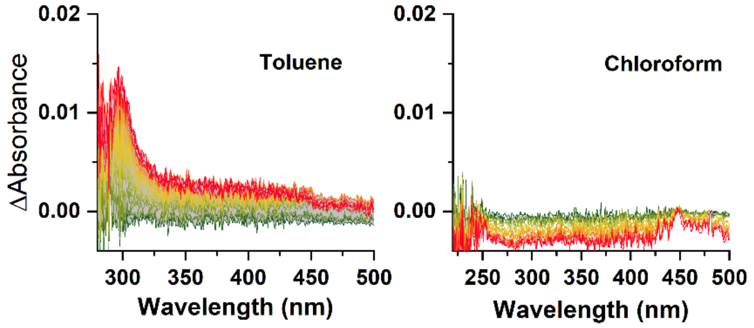

Figure S5. UV-visible solution spectra of $MAPbI_3$ after soaking in toluene and chloroform. Absorption peaks can be assigned to charge-transfer complexes of the solvents with iodine (toluene). The growing absorption peaks indicate degradation over time. Absorption of lead halide complexes (Hamill et al. ACS Energy Lett. 3, 92 (2018): PbI_2 PbI_3^- 368 nm, PbI_4^{2-} 410 nm) is not observable in these solutions.

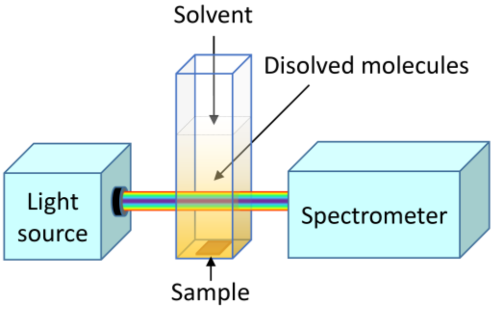

Figure S6. Illustration of UV-visible solution spectrum measurement set up. The solution is measured without illuminating the $MAPbI_3$ samples.

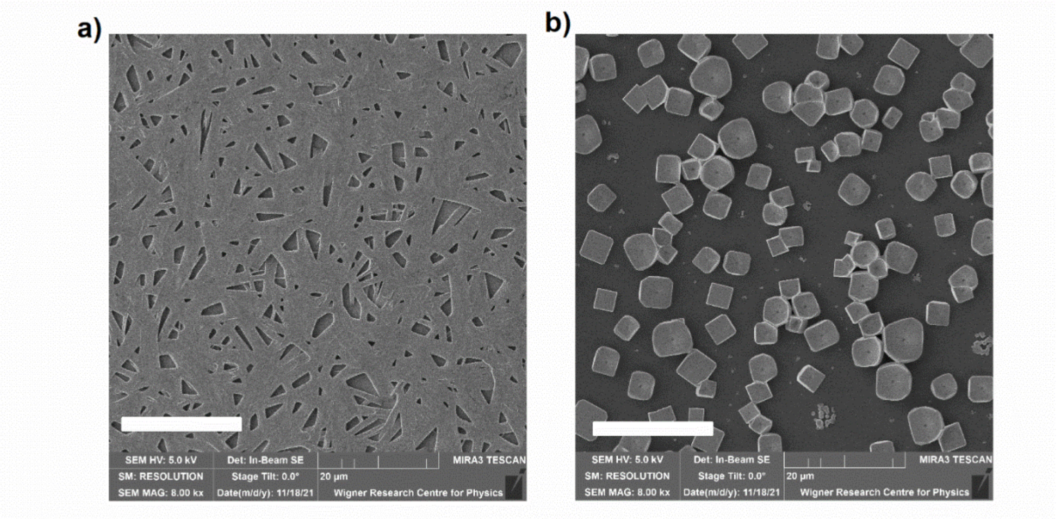

Figure S7. SEM images of $MAPbBr_3$ (a) and $MAPbI_3$ (b).

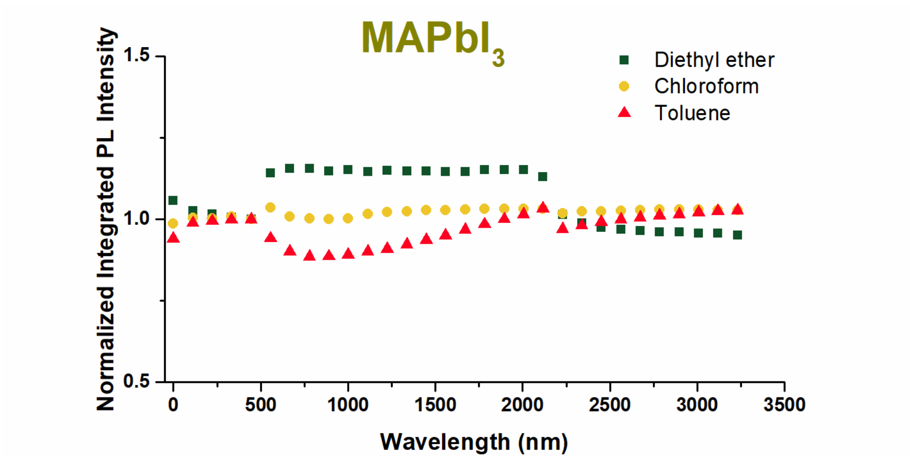

Figure S8. Enlarged PL intensity change of $MAPbI_3$ after exposure to low electron donor solvents. Chloroform very weakly interacts with $MAPbI_3$ surface, and interactions are more comparable to an inert response.

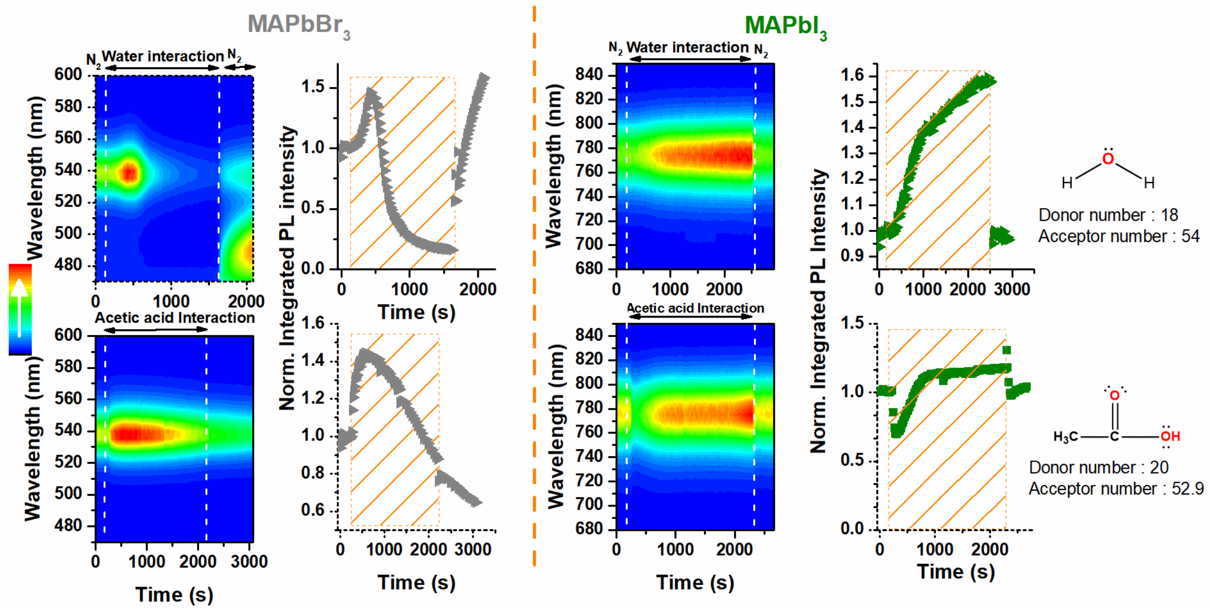

Figure S9. The effect of exposure of the surface of $MAPbBr_3$ and $MAPbI_3$ to acetic acid and water shown with higher resolution PL spectra. $MAPbBr_3$ and $MAPbI_3$ PL spectra are shown on the left and right side, and water and acetic acid effect on the spectra is shown in the first and second row, respectively. Different kinetic behaviour and the effect on the spectra of $MAPbI_3$ and $MAPbBr_3$ indicate that the chemical structure of the solvent molecules has an effect on the interaction mechanism even though the donor and acceptor numbers are similar. (Integration time is 0.2 seconds and wavelength range is shorter during the measurements compare to figure S1.)

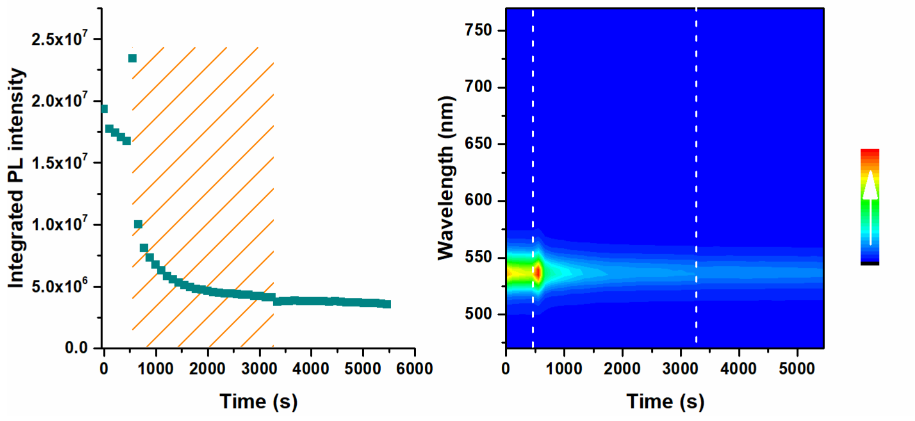

Figure S10. Extended exposure of $MAPbBr_3$ to acetic acid. The line graph of the integrated PL intensity and PL map of MAPbBr3 during the measurement is given on the left and right side of the graph. Acetic acid does not cause PL intensity quenching similar to water and reaches a steady state over time.

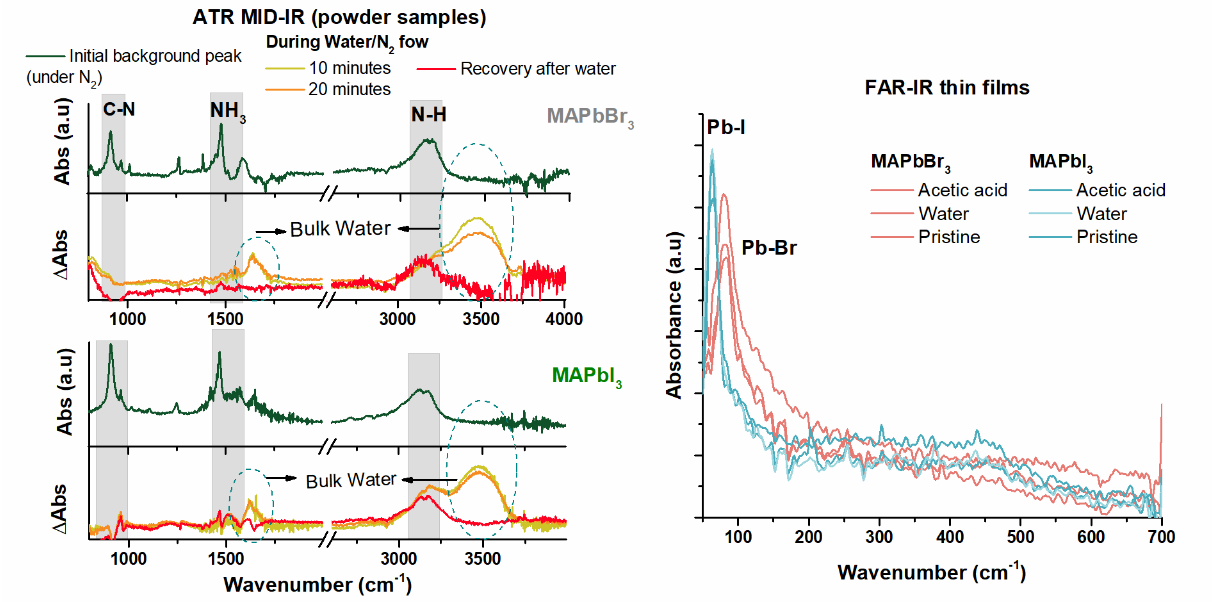

Figure S11. Infrared measurements of $MAPbBr_3$ and $MAPbI_3$. ATR mid-IR spectra depicted on the left are taken during and after water exposure. Far-IR spectra shown on the right are taken after the perovskite films were exposed to the indicated solvents. $MAPbI_3$ and $MAPbBr_3$ IR peaks are subtracted in ATR measurements for the clear observation of the water effect. Both mid- and far/IR measurement indicate the lack of reaction products.

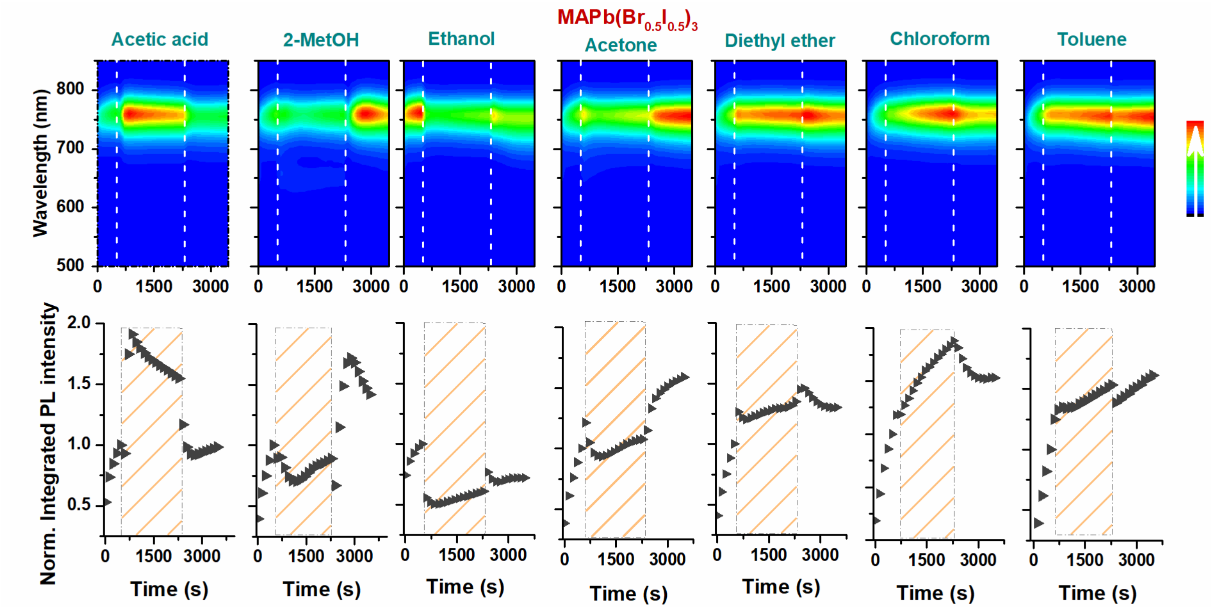

Figure S12. MAPb(Br$_{0,5}$I$_{0,5}$)$_3$ perovskite PL intensity changes under the moderate-low DN solvent exposure. White dashed lines in PL maps and orange patterned lines in line graphs represent the solvent exposure period (between 478-2272 seconds) in the measurement. (2-MetOH: 2-methoxyethanol)

Table S1. Properties of the selected solvents. [1,2]

Solvent	Donor Number (kcal/mole)	Acceptor Number (kcal/mole)	δ_{HB} $(MPa)^{1/2}$	Dielectric constant	Dipole Moment (D)
Dimethylformamide	26.6	16	11.3	36.71	3.79
2-methoxyethanol	20	39*	16.4	16.93	2.04
Water	18	54	42.3	80.10	1.87
Acetic Acid	20	52.9	13.5	6.20#	1.68
Ethanol	19.2	37.9	19.4	24.5	1.66
Acetone	17	12.5	7	24.4	2.69
Diethyl ether	19.2	3.9	5.1	4.33	1.3
Chloroform	4	23.1	5.7	4.81	1.15
Chlorobenzene	3.3	-	3.3	5.62	1.54
Toluene	0.1	-	2	2.38	0.31

*The value is according the Schmidt calculation and alcohol corrected calculation based on E_T (30) value[3], which is given as 52[4], equals to 38.4 from "AN: -39.69 + 1.503 E_T (30)" formula.

#Acetic acid forms cyclic dimers through hydrogen bod in pure form. [5]

REFERENCES

[1] V. Gutmann, *The Donor-Acceptor Approach to Molecular Interactions*; Springer US: Boston, MA, 1978.

[2] C. Laurence, J.-F. Gal, *Lewis Basicity and Affinity Scales: Data and Measurement*; Chippenham, Wiltshire, 2010.

[3] L. T. Evans, A simple solubility theory combining solubility parameter and Lewis acid-base concepts, Rochester Institute of Technology, 1988.

[4] C. Reichardt, *Chem. Rev.* 1994, *94*, 2319.

[5] O. W. Kolling, C. R. VanArsdale, *Trans. Kansas Acad. Sci.* 1965, *68*, 65.
